# Clinical outcomes and management of *CDKN2A/B* homozygously deleted IDH-mutant astrocytomas—a cohort study and patterns of care survey

**DOI:** 10.1093/nop/npaf045

**Published:** 2025-04-25

**Authors:** Alexander Yuile, Laveniya Satgunaseelan, Kimberley L Alexander, Subotheni Thavaneswaran, Hao-Wen Sim, Michael Krasovitsky, Benjamin Y Kong, Samuel Miller, Michael E Buckland, Maggie Lee, Grace Wei, Marina Kastelan, Mark Wong, Isabella Wilson, Angela Bayly, Winny Varikatt, Zarnie Lwin, Cassie Turner, Michael F Back, Nick Pavlakis, Joe Q Wei, Amanda Hudson, David L Chan, Helen R Wheeler, Adrian Lee

**Affiliations:** School of Medicine, University of Sydney, Australia; Medical Oncology Royal North Shore Hospital, Sydney, Australia; Department of Neuropathology Royal Prince Alfred Hospital, Sydney, Australia; School of Medical Sciences, Brain and Mind Centre, University of Sydney, Sydney, Australia; Neurosurgery Dept, Chris O’Brien Lifehouse, Sydney, Australia; Department of Neuropathology Royal Prince Alfred Hospital, Sydney, Australia; St Vincent’s Clinical School, University of New South Wales, Sydney, Australia; Kinghorn Centre St Vincent’s Hospital, Sydney, Australia; Garvan Institute of Medical Research, Sydney, Australia; Medical Oncology Dept, Chris O’Brien Lifehouse, Sydney, Australia; The Brain Cancer Group, North Shore Private Hospital, Sydney, Australia; Medical Oncology Royal North Shore Hospital, Sydney, Australia; Radiation Oncology Royal North Shore Hospital, Sydney, Australia; School of Medical Sciences, Brain and Mind Centre, University of Sydney, Sydney, Australia; Department of Neuropathology Royal Prince Alfred Hospital, Sydney, Australia; Department of Neuropathology Royal Prince Alfred Hospital, Sydney, Australia; The Brain Cancer Group, North Shore Private Hospital, Sydney, Australia; Medical Oncology Department Westmead Hospital, Sydney, Australia; Tissue Pathology & Diagnostic Oncology, ICPMR, Westmead; Tissue Pathology & Diagnostic Oncology, ICPMR, Westmead; Faculty of Medicine, University of Queensland, Australia; Royal Brisbane and Women’s Hospital, Queensland, Australia; Medical Oncology Royal North Shore Hospital, Sydney, Australia; Medical Oncology Royal North Shore Hospital, Sydney, Australia; Medical Oncology Royal North Shore Hospital, Sydney, Australia; School of Medicine, University of Sydney, Australia; Medical Oncology Royal North Shore Hospital, Sydney, Australia; Medical Oncology Royal North Shore Hospital, Sydney, Australia; Medical Oncology Royal North Shore Hospital, Sydney, Australia

**Keywords:** astrocytomas, *CDKN2A/B* deletions, IDH-mutant, outcomes

## Abstract

**Background:**

The use of *CDKN2A/B* deletions in the current WHO CNS classification (5th edition, 2021), has led to some ambiguity in patient management. Further clinical studies are required to fully ascertain the clinical impact of *CDKN2A/B* deletions in this patient cohort and their optimal management. To this end, we conducted a multi-center retrospective cohort study and patterns of care survey to explore real-world outcomes in *CDKN2A/B* homozygously (HoD) and heterozygously deleted (HeD) IDH mutant tumours.

**Methods:**

Demographic and clinical data were compiled for patients with IDH-mutant astrocytomas across multiple oncology centers and databases. Findings were supplemented with patterns of care survey circulated to Australian neuro-oncology centers and neuro-pathologists.

**Results:**

Patients were divided into the following cohorts: *CDKN2A/B* HoD (*n* = 12), *CDKN2A/B* HeD (*n* = 16) and non-*CDKN2A/B* deleted (*n* = 41). The *CDKN2A/B* HoD cohort had a median overall survival (OS) of 4.57 years, however, median OS was not yet reached for any of the other cohorts. The 5-year OS for the HoD cohort was % compared to 88% in the HeD cohort and 88% in the non-*CDKN2A* deleted cohort (Log-Rank *P = *.012). Respondents to our patterns of care survey preferred concurrent radiotherapy-temozolomide therapy followed by adjuvant temozolomide for *CDKN2A/B* HoD IDH-mutant astrocytomas regardless of the morphologic grade.

**Conclusion:**

This exploratory study supports the importance of molecular profiling in glioma diagnoses and the prognostic utility of current classification systems. However future research into the optimal therapy for these patients is needed in prospective studies.

**Key Points**

1. *CDKN2A/B* HoD in IDH-mutant astrocytomas confers a worse prognosis than non-*CDKN2A/B* deleted and HeD deleted IDH-mutant astrocytomas regardless of morphologic grade.
2. However, no statistically significant difference in overall survival was observed among *CDKN2A/B* HeD and non-*CDKN2A/B* deleted IDH-mutant astrocytomas.
3. Reported patterns of care support managing *CDKN2A/B* homozygous deleted *IDH* mutant astrocytomas with more intensive treatment strategies regardless of morphologic grade. However, this does not extend to *IDH* mutant astrocytomas with a heterozygous deletion.

**Importance of the Study**

Here we report on the largest *CDKN2A/B* HoD and HeD in IDH-mutant astrocytomas cohort in Australia. In addition, we are the first to conduct patterns of care survey regarding approaches to testing and treatment for *CDKN2A/B* HoD and HeD. Our findings help to further validate and refine the use of *CDKN2A/B* deletions (both HoD and HeD) in diagnosis, prognosis, and management.

Molecular phenotyping has become a crucial component in classifying gliomas and has a significant influence on prognosis.^1^ A prime example of this is mutations in the genes *isocitrate dehydrogenase1/2* (*IDH1/2*). The encoded enzymes convert alpha-ketoglutarate to *R*-2-hydroxygluturate (2-HG), which in turn drives oncogenesis and global epigenetic changes within the tumor.^2–4^ Since 2016, the World Health Organisation (WHO) Classification of Tumour of the Central Nervous System has used the presence/absence of *IDH1/2* mutations to dichotomize adult diffuse gliomas.^5^ In its most recent iteration (WHO CNS 2021),^6^ the CNS WHO classification recognizes gliomas with astrocytic and high-grade features without an *IDH1/2* mutation as IDH*-*wildtype glioblastoma (CNS WHO grade 4), as compared to gliomas with astrocytic features and an *IDH1/2* mutation as IDH-mutant astrocytomas (CNS WHO grade –4).^6^

IDH-mutant astrocytomas account for 80% of CNS WHO grade 2–3 gliomas and 5% of CNS WHO grade 4 gliomas^7,8^ and are pathologically distinct from IDH-wildtype glioblastomas. When compared with IDH-wildtype glioblastomas, patients with IDH-mutant astrocytomas are, on average, younger at diagnosis (aged 30–40 years) when compared to IDH-wildtype glioblastomas (aged 50 years and older). Furthermore, high-grade IDH-mutant astrocytomas have a more favorable prognosis compared to IDH-wildtype glioblastomas with median overall survival (OS) of 31 months and 15 months, respectively.^8^ Yet, not all IDH-mutant gliomas have a favorable course with multiple reports indicating that molecular heterogeneity can drive variable clinical outcomes and survival.^9^

Molecular markers implicated in this survival discrepancy within IDH*-*mutant gliomas are *cyclin dependant kinase inhibitor 2A* and/or *cyclin dependant kinase inhibitor 2B* (*CDKN2A/B*) deletions.^9^*CDKN2A* encodes for two proteins, p14 and p16; and *CDKN2B* encodes p15^10^ that have tumor suppressor roles through a variety of mechanisms including Rb-mediated cell cycle arrest, inhibition of angiogenesis, and indirect modulation of p53.^11–15^*CDKN2A* and *CKDN2B* are located on chromosome 9p21 and due to their close proximity, are often co-deleted.^16^ Monoallelic loss is termed heterozygous deletion (HeD), while biallelic loss is termed homozygous deletion (HoD).

Numerous retrospective studies have shown that *CDKN2A/B* HoD is associated with a poor prognosis in IDH-mutant astrocytomas with an OS limited to 3 years regardless of histologic grade.^17–20^ For this reason, the Consortium to Inform Molecular and Practical Approaches to CNS Tumour Taxonomy- Not Official WHO (cIMPACT-NOW) working group recommended the grading of IDH*-*mutant astrocytomas include the presence of *CDKN2A/B* HoD. Following this, the latest CNS WHO classification^6^ incorporated *CDKN2A/B* HoD into the grading of IDH-mutant astrocytic gliomas. The term IDH-mutant glioblastoma was replaced by *IDH-*mutant astrocytoma, CNS WHO grade 4. An IDH-mutant astrocytoma (by definition, 1p/19q non-codeleted) is classified as grade 4 based on morphology (necrosis or microvascular proliferation) or by the presence of *CDKN2A/B* HoD^6^ (see Figure 1). By contrast, the prognostic role of *CDKN2A/B* HeD in IDH-mutant astrocytomas lacks evidence, with variable reports on its impact on survival^21–24^ and uncertainty around the threshold for copy number variant (CNV) calling cut-offs. Thus, *CDKN2A/B* HeD has not been adopted in the current diagnostic criteria.^6,9^

**Figure 1.**
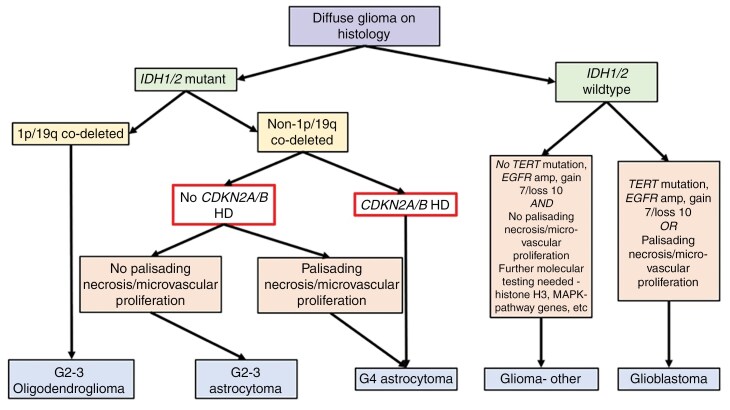
Flow diagram summarising WHO Classification of CNS Tumours (5th edition, 2021)^6^ and role of CDKN2A/B HOD. Note: gain 7/loss 10 refers to gain of chromosome 7 and loss of chromosome 10. Reproduced with permission from Yuile *et al*.^21^

The evolving CNS classification system has created some ambiguity and challenges in interpreting existing data, as the majority of IDH-mutant astrocytoma CNS WHO grade 4 were previously classified as grade 3 IDH-mutant astrocytoma or IDH-mutant glioblastoma under older nomenclature. Furthermore, there is no clear evidence regarding how these tumors should be treated clinically.^5,25^ Prior to the CNS WHO 2021, IDH-mutant glioblastomas were treated with concurrent radiotherapy and temozolomide followed by sequential temozolomide (e.g., the EORTC 26981-22981NCIC CE.3 protocol) and grade 3 IDH-mutant astrocytomas with radiotherapy alone followed by sequential temozolomide for 6-12 months (CATNON protocol).^26,27^ Moreover, there are limited prognostic studies that have adopted the current WHO classification for IDH-mutant astrocytomas.^21^ For instance, some reports describe *CDKN2A/B* HoD as a prognostic tool in combination with other molecular aberrations.^18,20,28^ In addition, the literature frequently describes the isolated effects of *CDKN2A* HoD without considering *CDKN2B* HoD,^19,21,29^ and the prognostic impact of *CDKN2A/B* HeD is yet to be resolved. In this report, we applied the updated WHO 2021 classification to identify patients diagnosed with IDH-mutant astrocytoma tumors with known *CDKN2A/B* status across Australia. By documenting the clinical characteristics and outcomes of this patient cohort, we aimed to describe treatment strategies and variations for optimal patient management.

## Materials and Methods

### Ethics

This study was conducted in accordance with the Declaration of Helsinki and approved by the Ethics Committee of Northern Sydney Local Health District (2020/ETH01474 and 2024/ETH00031).

### Patient Outcomes

*Patient cohort.*—Australian neuro-oncology centers and research databases were approached to determine if they had known *CDKN2A/B* deletion patients or tested for *CDKN2A/B* deletions in their glioma patients. Patients with IDH-mutant gliomas who had undergone molecular testing for *CDKN2A/B* deletion were identified from Royal North Shore Hospital, the Garvan Institute of Medical Research MoST Database, Royal Prince Alfred Hospital, Westmead Hospital, Royal Brisbane and Women’s Hospital and Sydney Brain Tumour Bank. Samples were only considered if the *CDKN2A/B* status was known from the initial surgical specimen. The deletion status of a recurrent sample could not be used to infer the deletion status of the primary resection, regardless of whether there was no intervening radiotherapy or systemic therapy. Data concerning patient demographics, the nature of the *CDKN2A/B* alteration, clinicopathologic factors, and OS were collected. Patients with known *CDKN2A/B* deletions were grouped into homozygous (HoD) and heterozygous (HeD) deletion cohorts.

For comparison, an index cohort was established comprising patients with IDH-mutant astrocytomas with confirmed presence of both *CDKN2A/B* genes. This index cohort was established from eligible patients identified during a record search across the aforementioned study sites.

*Assessment of molecular characteristics.*—All patients included in the dataset had IDH-mutant status confirmed through IDH1 R132H immunohistochemistry (IHC) or next-generation sequencing (NGS) (Illumina or IonTorrent sequencing platforms). To be eligible for inclusion, *CDKN2A/B* deletion status was identified by either NGS or fluorescence *in situ* hybridization (FISH) (Vysis CDKN2A/CEP 9 FISH Probe Abbott Laboratories, North Chicago, IL, USA) from tumor samples at the time of first diagnosis. The loss of MTAP can be used as a surrogate for *CDKN2A/B* HoD given the close proximity of the genes on chromosome 9p21(24). For this reason, patients with CDKN2A/B status assessed through MTAP IHC (Clone 2G4 monoclonal antibody; Taipei, Taiwan) were also included if CDKN2A/B deletion status was subsequently confirmed with FISH. Of these, nine had known CDKN2B status and seven of these harbored a synchronous CDKN2B homozygous deletion. CDKN2A HeD were present in 23% of cases (*n* = 16). Of these, 10 had known CDKN2B status and all 10 of these had synchronous CDKN2B HeD. Patients were excluded from either cohort if the *CDKN2A/B* deletion status could not be confirmed at diagnosis (ie, their molecular testing was only performed at recurrence).

### Patterns of Care Survey

An online survey was designed and approved by the Northern Sydney Local Health District Human Research and Ethics Committee. The survey questionnaire targeted medical oncologists and pathologists who treat adult brain tumors. Study data were collected and managed using REDCap electronic data capture tools hosted by the Northern Sydney Local Health District.^30,31^ Questions were aimed at assessing the respondents’ exposure to reports with *CDKN2A/B* alterations, testing practices, and management approaches to the WHO 2021 classification. Clinician questions were directed toward treatment, while pathologist questions were directed toward testing practices. We approached treatment centres that service population sizes sufficient for considerable experience in treating IDH-mutant astrocytoma patients including those with *CDKN2A/B* alterations. Surveys were distributed via email to clinicians, on a per-center basis, from a total of 16 centers across Australia. Surveys were distributed to pathologists through the Australian and New Zealand Society for Neuropathology (ANZSNP).

### Statistical Analysis

Patient cohorts were defined as *CDKN2A/*B HoD, HeD, and non-*CDKN2A/B* deleted IDH-mutant astrocytoma and summarized with descriptive statistics. The patient clinical factor differences among cohorts were described using analysis of variance (ANOVA) or Fisher’s exact test where appropriate. For the primary survival analysis, OS was compared among the HoD, HeD, and the non-*CDKN2A/B* deleted cohorts. A secondary pair-wise analysis among these cohorts was performed where the primary analysis achieved significance (*P <* 0.05). Given the exploratory nature of the analysis and limited cohort sizes, we did not correct for multiplicity. OS was calculated as the time between the date of the first surgery and the date of death. The median OS was estimated using Kaplan-Maier (KM) curve with comparisons conducted using the log-rank test. Median follow-up times were calculated using the reverse KM estimator.^32^

First-line treatment regimens for the *CDKN2A/B* HoD cohort are presented using descriptive statistics; no formal survival analysis was conducted among regimens due to insufficient events and low numbers. Given the low incidence of *CDKN2A/B* HoD, it was not feasible to perform a sample size calculation, and all available cases were included for analysis. All statistical analyses were conducted using Jamovi version 2.3.21 powered by R version 4.1,^33,34^ graphs, and survival curves were generated using GraphPad Prism version 9.4.1.

## Results

### Patient Outcomes

**Figure 2.**
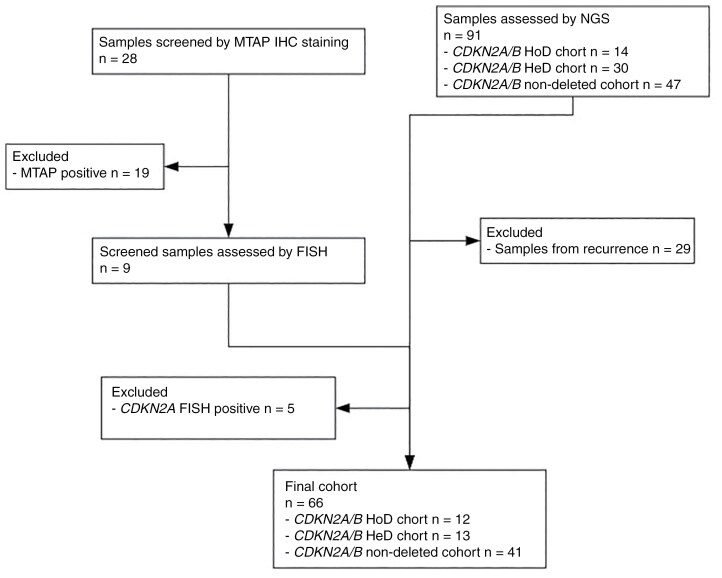
Consort diagram for cohort selection.

*Cohort characteristics.*—We identified 106 patients with IDH-mutant astrocytomas and known CDKN2A/B deletion status. Of these, 38% (*n* = 40) were excluded as the CDKN2A/B status was determined for a tumor recurrence and the initial resected tumor status was unknown. Therefore 66 patients were used in the analysis, being diagnosed between January 2007 and November 2022. Of these patients, *CDKN2A/B* deletion status was assessed by NGS for 94% of cases (*n* = 62). The remaining cases were identified through MTAP IHC screening. Out of a total of 28 cases stained for MTAP (*n* = 17 grade 3 cases and *n* = 11 grade 4 cases), 4 cases were considered as MTAP negative with five cases being borderline. All nine cases proceeded to FISH analysis which identified three cases with a CDKN2A/B HoD and one case with a CDKN2A/B HeD (see Figure 2). Basic patient demographics and tumor characteristics are presented in Table 1. Basic patient demographics and tumor characteristics are presented in Table 1.

**Table 1.** Patient demographics and tumor characteristics

	*CDKN2A/B* HoD astrocytomas *n* = 12	*CDKN2A/B* HeD astrocytomas *n* = 13	Non-*CDKN2A/B* deleted astrocytomas *n* = 41	*P-*value
Age (years) median (range)	32.5 (25-62)	36 (20-74)	34 (22-67)	.857^*^
Gender (female) *n* (%)	4 (33)	7 (54)	19 (46)	.644^**^
ECOG *n* (%)				
0	4 (36)	10 (77%)	21 (72)	**.032** ^**^
1	2 (18)	2 (15)	7 (24)	
2	3 (27)	0	1 (3)	
3	1 (9)	1 (8)	0	
4	1 (9)	0	0	
Unknown	1	0	12	
Morphologic grade *n* (%)				
2	0 (0)	0 (0)	14 (34)	**.006** ^**^
3	7 (58)	11 (85)	20 (49)	
4	5 (42)	2 (15.4)	7 (17)	
Ki-67 index median (range)	10.0 (4.8-40.0)	15 (5-50)	10.0 (1.0-90.0)	.737^*^
Unknown	4	2	5	
*MGMT* Promoter Methylation status (% of total tested)				
Unmethylated	0	3 (23)	6 (15)	.238^**^
Methylated	5 (42)	3 (23)	9 (22)	
Not performed/unknown	7	7	26	
Resection status				.407^**^
Gross total	3 (25)	4 (31)	17 (41)	
Non-gross total	5 (42)	2 (15)	9 (22)	
Unknown	2	7	15	
Lines of treatment				
0	2 (17)	0	3 (11)	.129^**^
1	5 (42)	9 (69)	19 (68)	
2	3 (25)	1 (8)	5 (18)	
3	2 (17)	2 (15)	0	
4	0	0	1 (4)	
5	0	1 (8)	0	
Unknown	0	0	23	

^*^ANOVA,

^**^Fisher’s exact test.

Characteristics were balanced among the cohorts except for morphologic grading, where grade 4 astrocytomas were most represented in the *CDKN2A/B* HoD cohort and ECOG status, with the *CDKN2A* HoD cohort having a higher proportion of patients with a poor performance status. We identified homozygous *CDKN2A* deletions in 18% of the total cases (*n* = 12). Of these, 9 had known *CDKN2B* status and 7 of these harbored a synchronous *CDKN2B* homozygous deletion. *CDKN2A* HeD were present in 20% of cases (*n* = 13). Of these, 9 had known *CDKN2B* status and all 9 of these had a synchronous *CDKN2B* HeD. No *CDKN2B* deletions (homozygous or heterozygous) occurred without a synchronous *CDKN2A* deletion. The remaining 41 IDH-mutant astrocytoma cases were confirmed to be *CDKN2A/B* replete and formed the non-*CDKN2A/B* deleted cohort.

*Survival analysis by deletion status.*—The median follow-up for the *CDKN2A/B* HoD cohort was 9.6 years and 5.73 years for the *CDKN2A/B* HeD cohort. The median follow-up for the non-*CDKN2A/B* deleted cohort was 1.3 years overall.

**Figure 3.**
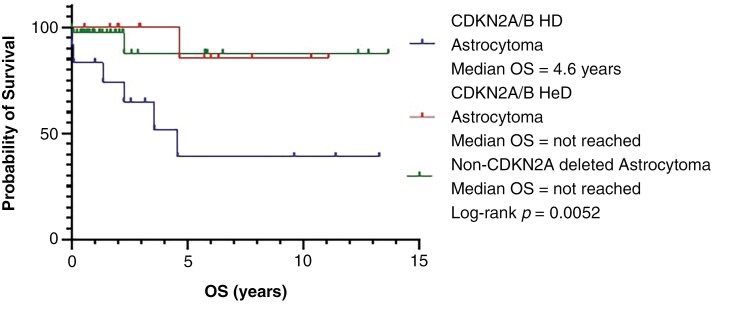
Overall survival of *CDKN2A/B* HoD vs HeD vs non-*CDKN2A/B* deleted IDH-mutant astrocytomas.

The CDKN2A/B HoD cohort had a median OS of 4.6 years with 6 deaths, however, median OS was not yet reached for the HeD cohort or the non-CDKN2A/B deleted cohort, with 2 deaths in each. We also evaluated overall survival between patients with a homozygous deletion in both CDKN2A and CDKN2B compared to those with only a CDKN2A deletion, however, no significant difference was found (Log-Rank *P* = 0.99). The 3-year OS for the HoD cohort was 65%, while it was 100% for HeD cohort and 88% for the non-*CKDN2A* deleted cohort. The 5-year OS for the HoD cohort was 39% compared to 86% in the HeD cohort and 88% in the non-*CDKN2A* deleted cohort. There was a significant difference in OS among the *CDKN2A/B* HoD and HeD and non-*CDKN2A* deleted cohorts (Log-Rank *P *= .0052) (see Figure 3). We then performed pair-wise analyses among cohorts and found a statistically significant difference in OS among the HoD and non-*CDKN2A/B* deleted cohorts (Log-Rank *P* = .010) and among the HoD and HeD cohorts (Log-Rank *P = *.017). There was no statistical difference in OS among the HeD cohort and the non-*CDKN2A/B* cohort (Log-Rank *P* = .787).

*CDKN2A/B homozygous deletions by treatment status.*— In the *CDKN2A/B* HoD cohort 17% (*n* = 2) were treated with concurrent radiotherapy and temozolomide followed by sequential temozolomide, 33% (*n* = 4) with radiotherapy alone followed by sequential temozolomide and 42% (*n* = 5) were treated with “other” regimens. The description of “other” varied, but included patients who did not proceed with any adjuvant therapy and patients receiving radiotherapy alone. We were unable to determine the treatment status of one patient.

### Patterns of Care Survey

*Background of respondents .*—Surveys were circulated between October 2022 and November 2022. We invited 16 neuro-oncology centers to participate and achieved a response rate of 81% (*n* = 13). Of these responses, 92% (*n* = 12) were completed by medical oncologists, with the remaining respondent being a radiation oncologist. Most respondents identified as being from a tertiary neuro-oncology or metropolitan center (77% and 15% respectively) and one respondent identified their center as rural. When respondents were asked how many astrocytic gliomas they see annually, 62% reported greater than 20 astrocytic gliomas a year, 23% see 15–20 per year and 15% see 9–11 cases. The median reported percentage of these astrocytic gliomas that were IDH-mutant was 20% (range 5%–40%).

The neuropathologist survey was distributed to 94 pathologists, with 14% (*n* = 13) responding. Most respondents (85%) identified as working in a tertiary neuro-oncology center, with one respondent from a metropolitan center and another from a private center. 55% of the respondents saw greater than 20 astrocytic gliomas per year, 31% saw 15–20 cases per year, and 8% see 12–14 and 9–11 cases per year. The median percentage of these cases being IDH-mutant astrocytomas was estimated as 8% (range 0%–30%) by respondents.

*Diagnostic trends.*—For the clinician group, 39% of respondents routinely request molecular diagnostics to determine the presence of *IDH1/2* mutations. In respondents who did not routinely request *IDH1/2* sequencing, all cited the patient’s age as a trigger for supplementary molecular testing of IDH1 R132H wildtype cases. Other determinants included MRI appearance (50%), duration of symptoms to diagnosis (13%), and an equivocal IDH1 R132H IHC finding (13%). The incorporation of *CDKN2A/B* HoD into the CNS WHO 2021 classification changed practice for the majority of respondents (62%), with approximately a third currently routinely requesting *CDKN2A/B* testing.

In the neuropathologist group, 23% perform molecular analysis for *IDH1/2* mutations routinely. In centers that do not routinely sequence tumors for *IDH1/2* mutations, 90% of respondents cited the patient’s age as a trigger for supplementary molecular testing of IDH1 R132H wild-type cases. Other parameters that prompt further testing included MRI appearance (30%) as well as a loss of ATRX expression determined by IHC and other morphological features atypical of glioblastoma (40%). Most (92%) respondents agreed that the addition of *CDKN2A/B* HoD into the CNS WHO 2021 classification of IDH-mutant astrocytoma changed their standard practice with 54% implementing routine testing to assess *CDKN2A/B* gene alterations. The methodology used to determine the *CDKN2A/B* status were predominantly DNA-based studies, that is, NGS (31%), methylation profiling (23%), SNP-assays (23%), and FISH analysis (31%), however, some used MTAP IHC to indirectly infer the *CDKN2A*/B status (8%).

**Figure 4.**
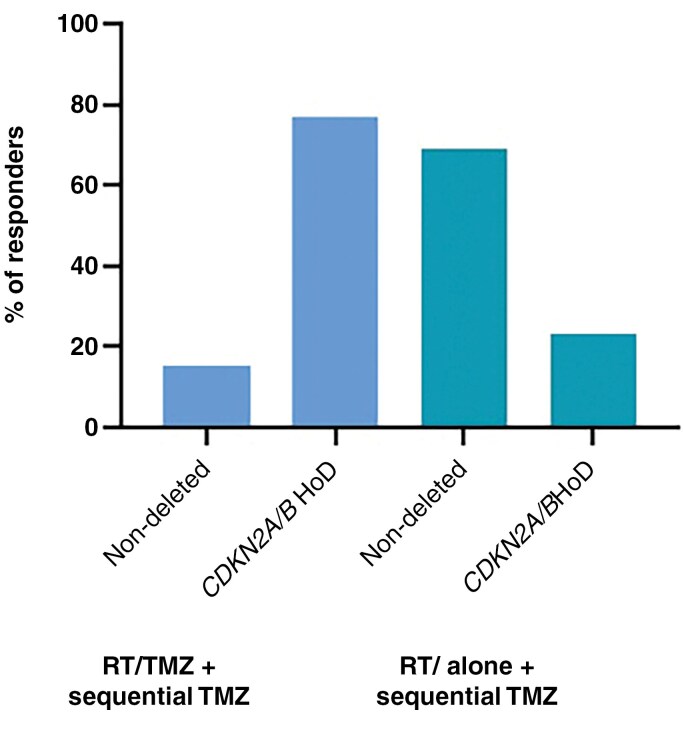
Proportion of centers who treat with concurrent radiotherapy/temozolomide (RT/TMZ) and sequential TMZ vs radiotherapy alone with sequential temozolomide.

*Management trends.*— Of the clinician group, 46% of centers stated they felt that knowledge of a *CDKN2A/B* HoD changes management and 23% stated they felt knowledge of a *CDKN2A/B* HeD changed management. Regarding treatment regimens, 69% of respondents treat morphologic grade 3 IDH-mutant astrocytomas with radiotherapy followed by sequential temozolomide, with the remainder using concurrent temozolomide-radiotherapy followed by sequential temozolomide. Of those centers that use radiotherapy alone with sequential temozolomide, 85% aim for 12 months of sequential temozolomide, and the remainder aim for 6 months. There was a 55% increase in centers that use the more intensive concurrent temozolomide-radiotherapy regimen in morphologic grade 3 astrocytomas with a *CDKN2A/B* HoD (Figure 4).

**Figure 5.**
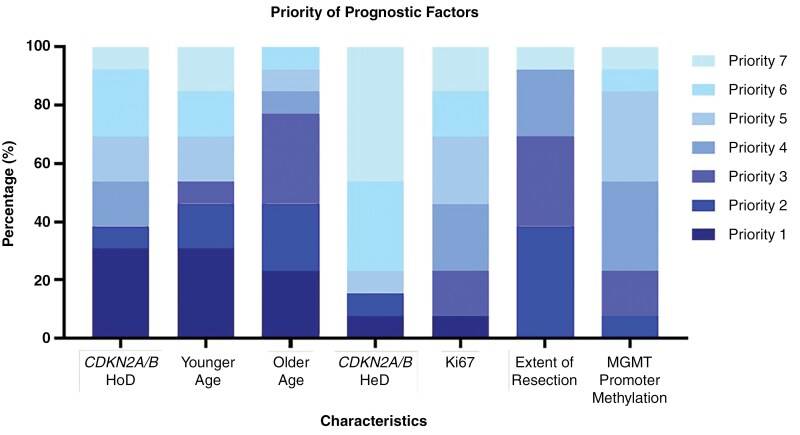
Distribution of Neuro-Oncology center weighting on a provided list of prognostic factors in glioma. Scale ranges from highest priority (Priority 1) to lowest priority (Priority 7).

Centers were asked to rank clinical, molecular, and histopathologic factors influencing management (1 being the most important and 7 being the least). Factors used included: *CDKN2A/B* HoD, *CDKN2A/B* HeD, Ki-67 index, age, *MGMT* promoter methylation status, and extent of resection (see Figure 5). The most commonly identified factors given the highest priority were both *CDKN2A/B* HoD and younger age (both 30.8%, *n* = 4). The lowest priority factor was *CDKN2A/B* HeD (46%, *n* = 6).

## Discussion

Here, we present real-world data regarding the impact of *CDKN2A/B* HoD and HeD patient outcomes in IDH-mutant astrocytomas, including the results of a nationwide patterns of care survey. We found that the CDKN2A/B HoD cohort had a significantly worse median overall survival when compared to the non-CDKN2A/B deleted cohort. This survival difference occurred despite including seven grade 4 IDH-mutant astrocytoma cases in the non-CKDN2A/B deleted cohort. There was no statistically significant difference between the *CDKN2A/B* HeD cohort and the non-*CDKN2A/B* deleted cohort survival. The patterns of care survey demonstrated that the presence of *CDKN2A/B* HoD impacts management, with clinicians opting for the more intensive concurrent radiotherapy-temozolomide regimen when it occurs in the setting of morphologic grade 3 *IDH*-mutant astrocytomas. We also observed that the majority of pathologists used DNA-based assays to confirm CDKN2A/B homozygous deletions. However, the method of DNA analysis was fairly even divided among NGS, methylation status, and FISH. This variation in molecular assessments likely reflects the differing access to NGS and whole methylome studies, with only a few centers offering this service in Australia. Access to FISH however is more widely available across major pathology centres. Our patterns of care survey also demonstrated the various approaches to screening for a CDKN2A/B homozygous deletion. Our experience suggests that recommendations regarding optimal testing for CDKN2A/B should take into account the resource level of the clinical laboratory, to ensure all patients can access CDKN2A/B testing in some form. We found that both FISH and NGS were acceptable methods of testing for CDKN2A/B deletion. It should be noted both forms of testing were performed in laboratories that had both clinically validated and accredited these tests.

Our findings are in keeping with the current literature. The high numbers of *CDKN2A/B* HoD astrocytomas with grade 4 morphology are consistent with other groups globally.^9,17–19,21^ Our finding that *CDKN2A/B* HoD had the worst prognosis of the three cohorts is also consistent with current data and supports the inclusion of *CDKN2A/B* assessment in IDH-mutant astrocytomas in the latest CNS WHO classification.^9,17–19,23^ However, we were unable to detect a significant difference in OS between the *CDKN2A/B* HeD and non-*CDKN2A/B* deleted cohorts. While the presence of a *CKDN2A* HeD was demonstrated not to impact downstream p16 transcript levels and, theoretically, have no substantive prognostic impact,^22^ there are reports to the contrary. For example, Kocakavuk *et al* documented a significantly shorter OS in *CDKN2A* HeD IDH-mutant astrocytomas compared to non-*CDKN2A/B* deleted cases.^24^ The reason for this conflicting result is unclear, however, it may be related to small sample sizes, population heterogeneity, and differences in testing methodologies across the various sites. Given that no patients were found to have a *CDKN2B* deletion without a co-occurring *CDKN2A* deletion, we cannot comment on the individual impact of *CDKN2B* deletions. However, our findings suggest that such an occurrence would be rare, which is in keeping with previous studies of gene deletion frequencies in the 9p21 region. In this report, we are the first to report patterns of care in *CDKN2A/B* HoD glioma patients across Australia. Our survey data indicates that *CDKN2A/B* HoD status has a major impact on the management approach, whereas *CDKN2A/B* HeD has minimal impact on clinical decision making. This is consistent with the current cIMPACT-NOW and CNS WHO 2021 recommendations.

We acknowledge the limitations of our study. The small number of *CDKN2A/B* HoD patients limits robust statistical analysis and increases the risk of bias, although there is the potential for future prospective data collection and collaborative data sharing to mitigate this. There was heterogeneity in the assessment of *CDKN2A/B* between NGS and FISH. However, given the multi-institutional nature of our study, variation in testing methods was unavoidable. Given the relative equivalence between both methods, this variation is likely to have minimal impact on study findings. Similarly, although MTAP IHC was used, given all samples with loss of MTAP were confirmed with FISH, this removes heterogeneity from this perspective. Additionally, the discrepancy in follow-up among the deleted and non-CDKN2A/B deleted groups suggests potential selection bias. This was due to retrospective testing being preferentially performed on patients with more aggressive tumor characteristics who had longer follow-up times. This differs from current practice, where systematic prospective testing is applied across all groups without focusing specifically on high-risk cases. Unfortunately, these factors were unavoidable given the retrospective nature of this study looking at a rare condition, however, this has been corrected to an extent with censuring on KM-analysis.. It should also be noted that there was a higher proportion of patients with *MGMT* promoter methylation in the *CDKN2A* HoD cohort. However, given the survival advantage associated with *MGMT* promoter methylation^35–37^ this difference could bias the findings towards no survival difference between the cohorts, suggesting that the true survival difference between CDKN2A HD and CDKN2A non-deleted patients may be even greater than reported.

Retrospective *CDKN2A/B* testing on archival samples at all involved centers would have improved our cohort numbers, however, its sensitivity would be limited by DNA degradation over time. In addition, information presented by the survey is prone to biases such as recall bias which may limit data interpretation. Survey participation was also lower than anticipated. As *CDKN2A/B* testing becomes more routine in clinical practice, we anticipate the expansion of our cohort over the next few years.

In conclusion, within the limits of small sample size, this exploratory study supports the prognostic utility of current classification systems and flags the need for further study of treatment implications in this area. Our patterns of care survey demonstrated a clear preference for concurrent chemoradiation with adjuvant temozolomide therapy for *CDKN2A/B* HoD IDH-mutant astrocytomas regardless of the morphologic grade. Due to expected small numbers, prospective multinational collaborations are required to build on this initial retrospective project to further establish the prognostic utility of *CDKN2A/B* HoD and *CDKN2A/B* HeD and its role in optimal clinical management.

## Data Availability

Data can be made available, when in keeping with ethics approval, by contacting the the corresponding author- details above.
